# PET/CT Imaging of ^89^Zr-N-sucDf-Pembrolizumab in Healthy Cynomolgus Monkeys

**DOI:** 10.1007/s11307-020-01558-w

**Published:** 2020-10-26

**Authors:** Wenping Li, Yuchuan Wang, Daniel Rubins, Idriss Bennacef, Marie Holahan, Hyking Haley, Mona Purcell, Liza Gantert, SuChun Hseih, Michael Judo, Wolfgang Seghezzi, Shuli Zhang, Elly L. van der Veen, Marjolijn N. Lub-de Hooge, Elisabeth G.E. de Vries, Jeffrey L. Evelhoch, Michael Klimas, Eric D. Hostetler

**Affiliations:** 1 Translational Biomarkers, Merck & Co., Inc., WP 44D, 770 Sumneytown Pike, West Point, PA 19486 USA; 2 PPDM Bioanalysis, MRL South San Francisco, 213 East Grand Blvd, South San Francisco, CA 94080 USA; 3 Department of Medical Oncology, University Medical Center Groningen, University of Groningen, Hanzeplein 1, 9713 GZ Groningen, The Netherlands

**Keywords:** PD-1-positive immune cells, Positron emission tomography (PET) imaging, ^89^Zr-N-sucDf-pembrolizumab, Cynomolgus monkeys, Mesenteric lymph nodes, Spleen and tonsils, Standardized uptake values (SUV_mean_)

## Abstract

**Purpose:**

Programmed cell death-1 receptor (PD-1) and its ligand (PD-L1) are the targets for immunotherapy in many cancer types. Although PD-1 blockade has therapeutic effects, the efficacy differs between patients. Factors contributing to this variability are PD-L1 expression levels and immune cells present in tumors. However, it is not well understood how PD-1 expression in the tumor microenvironment impacts immunotherapy response. Thus, imaging of PD-1-expressing immune cells is of interest. This study aims to evaluate the biodistribution of Zirconium-89 (^89^Zr)-labeled pembrolizumab, a humanized IgG4 kappa monoclonal antibody targeting PD-1, in healthy cynomolgus monkeys as a translational model of tracking PD-1-positive immune cells.

**Procedures:**

Pembrolizumab was conjugated with the tetrafluorophenol-N-succinyl desferal-Fe(III) ester (TFP-N-sucDf) and subsequently radiolabeled with ^89^Zr. Four cynomolgus monkeys with no previous exposure to humanized monoclonal antibodies received tracer only or tracer co-injected with pembrolizumab intravenously over 5 min. Thereafter, a static whole-body positron emission tomography (PET) scan was acquired with 10 min per bed position on days 0, 2, 5, and 7. Image-derived standardized uptake values (SUV_mean_) were quantified by region of interest (ROI) analysis.

**Results:**

^89^Zr-N-sucDf-pembrolizumab was synthesized with high radiochemical purity (> 99 %) and acceptable molar activity (> 7 MBq/nmol). In animals dosed with tracer only, ^89^Zr-N-sucDf-pembrolizumab distribution in lymphoid tissues such as mesenteric lymph nodes, spleen, and tonsils increased over time. Except for the liver, low radiotracer distribution was observed in all non-lymphoid tissue including the lung, muscle, brain, heart, and kidney. When a large excess of pembrolizumab was co-administered with a radiotracer, accumulation in the lymph nodes, spleen, and tonsils was reduced, suggestive of target-mediated accumulation.

**Conclusions:**

^89^Zr-N-sucDf-pembrolizumab shows preferential uptake in the lymphoid tissues including the lymph nodes, spleen, and tonsils. ^89^Zr-N-sucDf-pembrolizumab may be useful in tracking the distribution of a subset of immune cells in non-human primates and humans.

**Trial Registration:**

ClinicalTrials.gov Identifier: NCT02760225

**Supplementary Information:**

The online version contains supplementary material available at 10.1007/s11307-020-01558-w.

## Introduction

The field of immuno-oncology has expanded rapidly with the approval of therapies that target immune checkpoints, which increase T cell activity against cancer cells in the tumor microenvironment and enable tumor cells to escape immune response [1]. Antibody-based interventions targeting programmed cell death protein 1 (PD-1) on T lymphocytes and its principal ligand (PD-L1) on tumor cells appear to restore immune function in the tumor microenvironment and have produced significant antitumor activity with considerably less toxicity than conventional chemotherapy [2]. Such antibodies usually have high antigen specificity, which can be repurposed as imaging agents to selectively bind to targets to study changes in immune cells in pathologies like autoimmunity, infection, and cancer [3–5]. The reuse of therapeutic antibodies for imaging purposes reduces translational costs since the safety profile of the antibody is well defined and the agent is already available under conditions suitable for clinical applications. Molecular imaging with positron emission tomography (PET) using radiolabeled antibodies or antibody fragments (immuno-PET) allows non-invasive visualization of tumors, characterization, and quantification of the biological characteristics of cells and tissues in patients. It also may support optimal medical therapy for patients with cancer [6].

^89^Zr-radiolabeled antibodies for immune checkpoints such as PD-1 enable serial non-invasive imaging and quantification of the distribution of PD-1-expressing immune cells in cancer patients [7, 8]. The long half-life of ^89^Zr (78.4 h) allows imaging of a radiolabeled antibody several days after its injection and allows for improved image contrast due to decreased background signal. As a residualizing isotope, ^89^Zr remains inside cells if the antibody-antigen complex internalizes, allowing activity to accumulate and concentrate in tumors. In contrast, non-localized activity clears from the body, ultimately resulting in high-contrast images [9]. The PD-1-targeting immune checkpoint inhibitor pembrolizumab has been radiolabeled with ^89^Zr for PET imaging in rodent models of cancer [10, 11]. Although these studies evaluated the biodistribution and kinetics of this tracer, it is a challenge to directly compare the different studies and translate the results to a clinical setting. Some studies have used antihuman antibodies and human tumor xenografts, while others used antimouse antibodies. Additionally, different tumor cell lines and different mouse strains, with varying ages, were used. Furthermore, one important limitation is that preclinical investigation of antibody biodistribution in rodent models greatly varies from the observed kinetics in human patients [12]. An imaging study in large animal species, such as healthy cynomolgus monkeys with close genetic homology to humans could increase the understanding of the biodistribution of ^89^Zr-radiolabeled pembrolizumab. To our knowledge, this is the first study to investigate the pharmacokinetics, distribution, and specific binding of radiolabeled pembrolizumab in cynomolgus monkeys. This study reports the *in vivo* evaluation of ^89^Zr-N-sucDf-pembrolizumab in healthy cynomolgus monkeys as a preliminary study of biodistribution and clearance, investigating the translatability of PET using ^89^Zr-radiolabeled antibody in patients. PD-1 plays a critical role in CD8 T cell exhaustion in chronic infections and has potential applications in the treatment of chronic infections [4, 5]; ^89^Zr-N-sucDf-pembrolizumab may be of interest to study autoimmunity and infection in monkeys.

## Materials and Methods

### Reagents, Antibody, and Radiochemical

A human monoclonal IgG4 antibody pembrolizumab was obtained from MSD, Netherlands. Tetrafluorphenol-N-succinyl desferal-Fe(III) (TFP-N-sucDf) was purchased from ABX (Advanced Biochemical Compounds GmbH, Radeberg, Germany). Sodium carbonate (Na_2_CO_3_) and gentisic acid (2,5-dihydroxybenzoic acid) were obtained from Acros Organics (Morris Plains, NJ, USA); 1.0 M HEPES buffer was obtained from Invitrogen (Vilnius, Lithuania). Ethylenediaminetetraacetic acid (EDTA), triaminepentaacetic acid (DTPA), and 10 M phosphate-buffered saline (PBS) were purchased from Sigma-Aldrich (St. Louis, MO, USA). ^89^Zr (~ 1.48 GBq/ml of 1.0 M oxalic acid) was provided by Washington University (Saint Louis, MO, USA).

Supplies used in this process are PD10 size-exclusion chromatography column (GE Healthcare, Piscataway, NJ, USA), size-exclusion chromatography (SEC) column (Waters Acquity UPLC Protein BEH SEC, 200 A, 1.7 μm 4.8 × 150 mm), radio-thin layer chromatography (radio-TLC) plate (Biodex, Shirley, NY, USA), and pH test strips. Eluent for the HPLC was 10 mM ammonium acetate, pH 6.8. ^89^Zr radiolabeling yield and purity were checked using radio-TLC plate (eluent 50 mM DTPA pH 7). Radio-TLC plates were imaged, and analysis was performed with an image reader (Bioscan, Washington DC, USA). All activity measurements were performed in a dose calibrator (CRC-15PET Capintec, Inc., Ramsey, NJ, USA). Size-exclusion high-performance liquid chromatography (SEC-HPLC) was performed on an Agilent 1200 LC system (Agilent Technologies, Palo Alto, CA, USA) equipped with a binary pump (0.7 ml/min), an auto-injector, a diode array detector set to 280 nm, and a radioactive detector (LabLogic, Tampa, FL, USA). The size-exclusion chromatography column was equilibrated with HPLC eluent. The HPLC retention time of the product was 2.7 min.

### Preparation of N-sucDf-Pembrolizumab

Pembrolizumab was provided as a powder (50 mg) in a sterile vial. Buffer exchange and conjugation were performed using a previously reported method in the radiopharmacy unit of the department of Nuclear Medicine and Molecular Imaging, University Medical Center Groningen, The Netherlands [13–15]. N-suc-desferal-TFP ester was used as a chelator, because of extensive experience with this chelator, including rapid set up of quality control and subsequent translation to a GMP compliant production method. In short, buffer exchange for NaCl 0.9 % (Braun) was performed using a Vivaspin-2 concentrator (30 kDa) with a polyethersulfone filter (Sartorius, Goettingen, Germany). N-suc-desferal-TFP ester solution was added to pembrolizumab solution in an antibody:chelator molar ratio of 1:2 (400 nmol mAb per 800 nmol chelator, corresponding to 60 mg antibody). Fe(III) was removed from the intermediate N-sucDf-pembrolizumab by addition of EDTA at pH 4.0–4.5. Following 30 min incubation, the reaction mixture was purified by centrifugation (10 min) with a Vivaspin-2 filter. N-sucDf-pembrolizumab was collected after sterile filtration. The final chelator:antibody ratio, presence of aggregates, and concentration of N-sucDf-pembrolizumab were determined by SEC-HPLC. In each batch, N-sucDf-pembrolizumab was diluted to a concentration of 10.00 mg/ml and aliquoted in portions of 2.50 mg per vial and stored at −80 °C.

### Radiosynthesis of ^89^Zr-N-sucDf-Pembrolizumab

The desired volume (~ 40 μl, 110–120 MBq) of ^89^Zr oxalic acid solution was pipetted into a reaction vial and diluted with HEPES (300 μl). Twenty microliters of 2 M Na_2_CO_3_ were added, and the reaction vial was incubated for 1 min. The pH of the solution was adjusted to 7.5 by further addition of 2 M Na_2_CO_3_ (7 μl). N-sucDf-pembrolizumab (2.50 mg, 250 μl) was added, and the reaction mixture vial was incubated in a ThermoMixer (Eppendorf, Hauppauge, NY, USA) at 3000 g at room temperature. After 60 min, 50 mM DTPA (5 μl) was added. Radiochemical yield was determined by radio-TLC of a 2 μl aliquot. The reaction mixture was transferred onto a PD10 column followed by 1 ml of the gentisic acid (2.50 mg/ml in PBS prepared on the day of the radiosynthesis), and mobile phase. The eluate was discarded. A second fraction of the gentisic acid (1 ml) was eluted, and the eluate discarded. Finally, 1.5 ml of gentisic acid was transferred onto the column, and the eluate containing ^89^Zr-N-sucDf-pembrolizumab was collected and diluted with saline (1 ml).

### PET Imaging Study in Cynomolgus Monkeys

PET imaging was conducted at Merck & Co., Inc. (West Point, PA, USA) under the guiding principles of the American Physiological Society and the Guide for the Care and Use for Laboratory Animals published by the US National Institutes of Health (NIH publication No. 85-23, revised 1985) and was approved by the Institutional Animal Care and Use Committee at Merck & Co., Inc. (West Point, PA, USA). Four cynomolgus monkeys with no previous exposure to humanized monoclonal antibodies were used. Each monkey was initially sedated with ketamine (10 mg/kg intramuscular (im)), induced with propofol (5.00 mg/kg intravenously (iv)), intubated, and respired at ~ 10 cc/breath/kg and 23 respirations per minute. For each scan, the anesthesia was maintained with propofol (~ 0.40–0.60 mg/kg/min) for the duration of the PET study. Body temperature was maintained with circulating water heating pads, and temperature, SpO_2_, end-tidal CO_2_ were monitored. The animal was positioned on the bed of a Biograph PET/CT (Siemens, Knoxville, TN, USA). The body was placed in the center of transaxial field of view (FOV). Three monkeys received a tracer-only dose of 8.9–18.2 MBq of ^89^Zr-N-sucDf-pembrolizumab (0.177–0.301 mg dose of conjugated antibody) by saphenous vein infusion over 5 min on day 0. An additional monkey received 0.210 mg/kg pembrolizumab before tracer injection of ^89^Zr-N-sucDf-pembrolizumab (12.9 MBq, 0.255 mg) on day 0. Whole-body PET data were first collected at day 0, a few minutes after ^89^Zr-N-sucDf-pembrolizumab administration, and then on days 2, 5, and 7 following the initial scan, using the same scan protocol. Each whole-body PET scan consisted of 7 bed positions, with a 10-min emission acquisition per bed on day 0, and 20 min/bed on later days. Whole-body CT scans were also acquired at each time point, for PET attenuation and scatter corrections, as well as facilitating anatomical delineation of the regions-of-interest. Iterative reconstructions were performed for all PET data using the same set of manufacture-recommended reconstruction parameters. The image analysis software package PMOD (v3.8, PMOD Technologies LLC, Zürich, Switzerland) was used for quantitative analysis of the regions of interest (ROIs). ROIs were manually drawn, referencing both PET and CT data at each time point, including the blood, spleen, liver, lymph node, kidney, lung, muscle, brain, stomach, heart, thymus, tonsil, and small intestines. Subsequently, the image-derived biodistributions over the course of the imaging study (0 to 7 days post-injection) were calculated as standardized uptake values (SUV_mean_, tissue activity (MBq/cm^3^)/(injected dose (MBq)/body weight (g)) in the above ROIs for all imaging data sets.

Before and after each scan, whole blood samples (~ 1 ml each) were collected in plasma tubes and centrifuged, and the supernatants were isolated to determine the average plasma concentration of pembrolizumab at each PET time point. The antibody concentration in cynomolgus monkey plasma was measured using an electrochemiluminescent (ECL)-based ligand-binding assay on the Meso Scale Discovery (MSD) platform. The assay used sheep antihuman immunoglobulins (the binding site) as capture and sulfoTAG Mouse antihuman IgG (CH2 domain) (Thermo Scientific) as detection reagent. The plasma samples were diluted with assay buffer (DPBS [pH 7.4] containing 0.5 % bovine serum albumin, 0.05 % Tween-20, and 0.25 % 3-[(3**-**cholamidopropyl)dimethylammonio]-1-propanesulfonate hydrate (CHAPS), 5 mM EDTA and 0.35 M NaCl). They were captured on MSD multi-array high-binding plates coated with the sheep antihuman immunoglobulins, at room temperature for 1 h. After incubation, the plates were washed with wash buffer (DPBS [pH 7.4] with 0.05 % Tween-20), then detection reagent was added, and the plate was incubated as previously described [16]. After washing and addition of the read buffer, the plates were read by the MSD SECTOR Imager 6000, and the data was analyzed by MSD workbench software. The assay had a lower limit of quantitation (LLOQ) value of 0.003 μg/ml in plasma samples.

For dosimetry analysis, ROIs were drawn for the critical organs using the software VivoQuant (Invicro, Boston, MA, USA), and CT/PET images to guide region placement. The total amount of radioactivity contained in each organ was determined in units of becquerels (Bq), or expressed as a percentage of the total injected dose (% ID) by dividing the total decay-corrected activity in the ROI by the net injected dose. For each organ, the number of disintegrations per unit activity administered (Bq-hr/Bq) was obtained by calculating the area under the curve of the non-decay-corrected TACs using trapezoidal integration. All absorbed radiation doses were calculated using the OLINDA/EXM v1 software [17].

## Results

### Conjugation and Radiosynthesis of ^89^Zr-N-sucDf-Pembrolizumab

Pembrolizumab was conjugated with N-suc-desferal-TFP ester *via* an unmodified lysine side chains of pembrolizumab, resulting in a chelator: antibody ratio of 1.4:1 (Supplementary Figure 1). There were less than 5 % aggregates detected in the solution of conjugated antibody. Sixty minutes of incubation of N-sucDf-pembrolizumab with ^89^Zr solution resulted in an overall yield > 95 % and a radiochemical purity of ≥ 95 %. After PD10 column purification, the final radioactive concentration was 27–29 MBq/ml, and the molar activity was of 7–8 MBq/nmol.

### Animal Information

The animal characteristics, as well as monoclonal antibody doses for imaging, are summarized in Table 1. Four monkeys aged 7 to 11 years were included. Each animal received a dose of 8.9–18.2 MBq ^89^Zr-N-sucDf-pembrolizumab (0.177–0.301 mg dose of conjugated antibody). Animal 4 got an infusion of 0.210 mg/kg unlabeled pembrolizumab before the ^89^Zr-N-sucDf-pembrolizumab administration.

**Table 1.** Characteristics of cynomolgus monkeys studied

	Animal 1	Animal 2	Animal 3	Animal 4
Age	10.5	11.0	7.5	9.0
Body weight	9.00 kg	9.22 kg	6.82 kg	5.88 kg
Gender	Male	Male	Female	Female
Injected dose of ^89^Zr-N-sucDf-pembrolizumab	8.9 MBq (0.177 mg)	13.4 MBq (0.257 mg)	18.2 MBq (0.301 mg)	12.9 MBq (0.255 mg)
Injected pembrolizumab dose	0	0	0	1.240 mg^a^
Total injected protein dose	0.020 mg/kg	0.028 mg/kg	0.044 mg/kg	0.254 mg/kg

^a^Pembrolizumab was intravenously infused for 30 min

### Cynomolgus Monkey PET Imaging

**Fig. 1.**
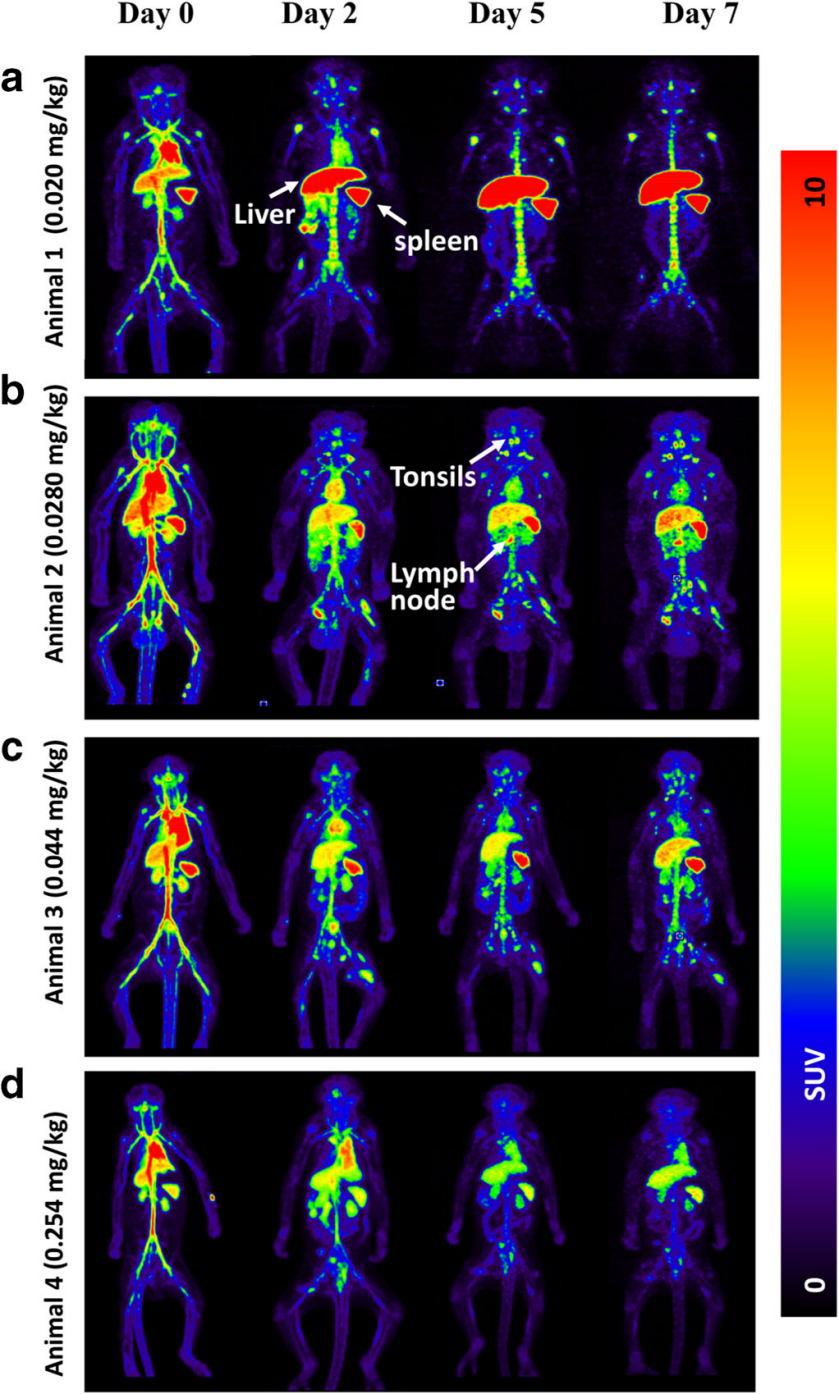
Representative PET images of ^89^Zr-N-sucDf-pembrolizumab in cynomolgus monkeys. Cynomolgus monkeys were intravenously injected with tracer only or tracer co-injected with pembrolizumab at day 0. PET scans were acquired at days 0, 2, 5, and 7 following injection. Image-derived standardized uptake values (SUV_mean_) were quantified by region of interest (ROI) analysis. White arrows indicate uptake in the liver, spleen, tonsils, and mesenteric lymph node (other lymph nodes observed, but not shown in these images). (a) Animal 1 tracer-only 0.020 mg/kg. (b) Animal 2 tracer-only 0.028 mg/kg. (c) Animal 3 tracer-only 0.044 mg/kg. (d) Animal 4 co-administration of 0.210 mg/kg pembrolizumab.

**Fig. 2.**
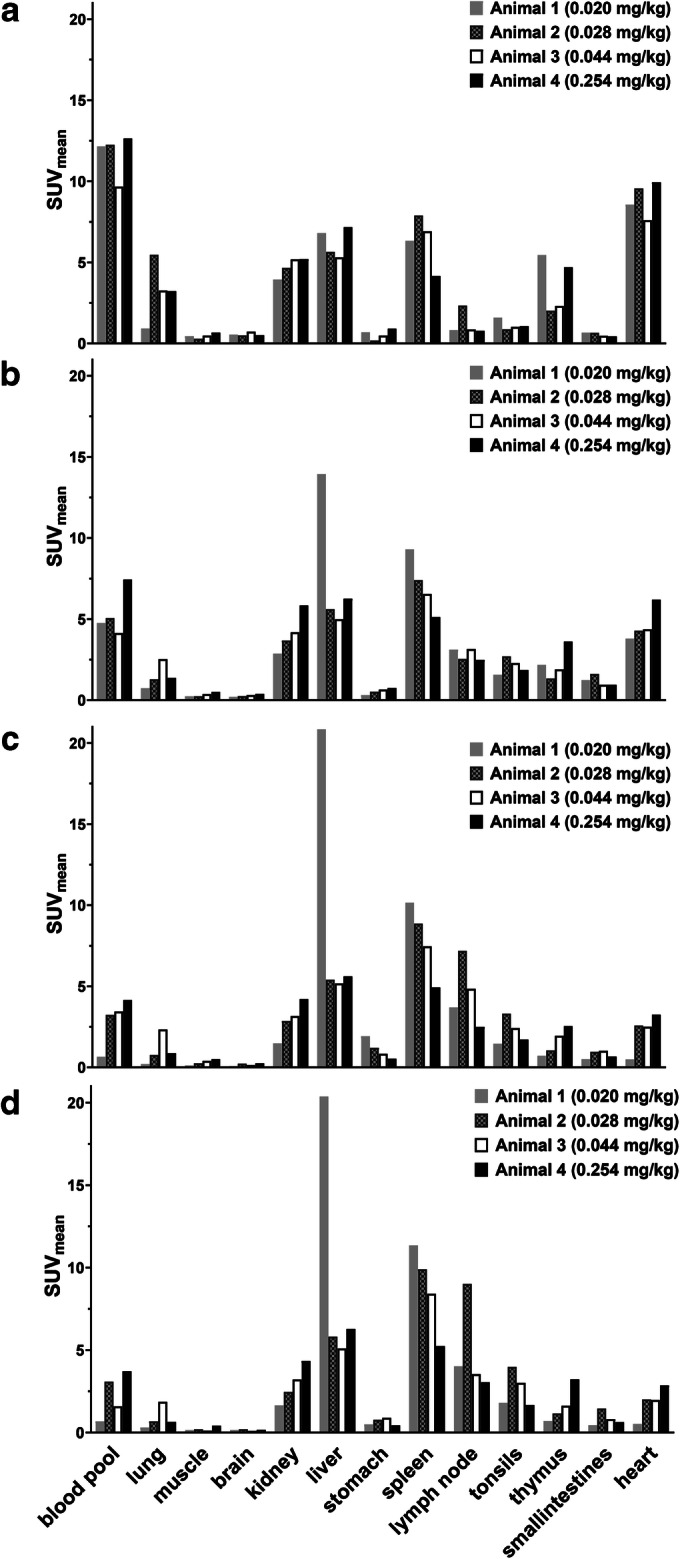
PET/CT image-derived radioactivity accumulation of ^89^Zr-N-sucDf-pembrolizumab in cynomolgus monkeys. Image-derived standardized uptake values (SUV_mean_) were quantified by region of interest (ROI) analysis following injection of ^89^Zr-N-sucDf-pembrolizumab. (a) At day 0, (b) at day 2, (c) at day 5, (d) at day 7.

**Fig. 3.**
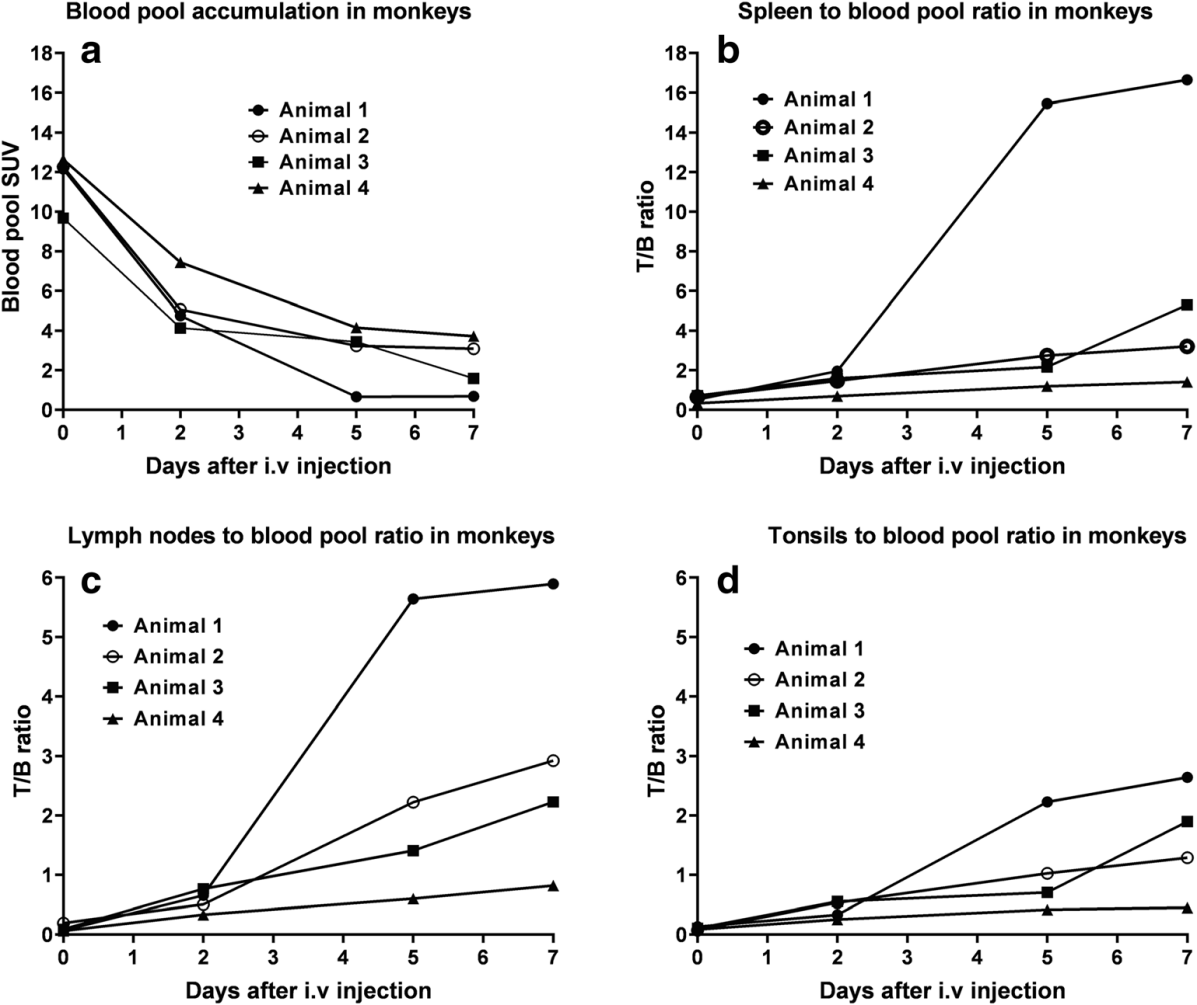
PET/CT image-derived radioactivity accumulation of ^89^Zr-N-sucDf-pembrolizumab determined as tissue to blood pool (T/B) ratio in cynomolgus monkeys. **a** Standardized uptake value (SUV_mean_) in blood pool. **b** SUV_mean_ ratio of spleen to blood pool. **c** SUV_mean_ ratio of lymph nodes to blood pool. **d** SUV_mean_ ratio of tonsils to blood pool.

Representative PET images in monkeys following ^89^Zr-N-sucDf-pembrolizumab injection at various time points are shown in Fig. 1. PET images were collected at days 0, 2, 5, and 7 after the injection of the radio-immunoconjugate. At the earliest time point (< 2 h), PET imaging showed tracer distribution into the primary and secondary lymphoid organs, including the bone marrow, spleen, tonsils, thymus, and lymph nodes. ^89^Zr-N-sucDf-pembrolizumab PET also showed elevated distribution in the heart, liver, and kidney, while accumulation was low in muscle and brain (Fig. 2). Over the course of 7 days, the background uptake (in the blood pool, lung, muscle, and brain) decreased for all monkeys independent of the total injected dose of protein. Levels of ^89^Zr-N-sucDf-pembrolizumab in the blood were the highest at 0.5 h (SUV_mean_ range 9.7–12.6) in all animals and gradually decreased (SUV_mean_ range 0.7–3.7) by day 7 (Fig. 3a). Tracer activity in blood was increased by co-administration of unlabeled pembrolizumab (animal 4). The lymphoid organs including the spleen, mesenteric lymph nodes, and tonsils showed a dose-dependent increase of the tissue to blood pool (T/B) ratio over time in each monkey (Fig. 3b, d). Day 7 post-tracer injection, animal 1 showed the highest ^89^Zr-N-sucDf-pembrolizumab T/B ratio in the spleen (16.6), mesenteric lymph nodes, (5.9), and tonsils (2.6). Animal 4, which received 10-fold excess unlabeled pembrolizumab, showed much lower ratios of T/B in lymphoid tissues at all time points examined. In contrast, non-lymphoid tissues showed similar T/B ratios to those observed in animals 1–3 (Figs. 2 and 3). At day 7, tracer distribution in the spleen (1.4), mesenteric lymph nodes (0.8), and tonsils (0.4) was low. Consistent with these results, the spleen uptake of ^89^Zr-N-sucDf-pembrolizumab was reduced by approximately 50 % with additional administration of 0.21 mg/kg pembrolizumab. This resulted in a decrease in the spleen radioactivity accumulation to SUV_mean_ of 5.24 in animal 4 at day 7 imaging point compared with SUV_mean_ of 11.35 in animal 1. Uptake in other lymphoid organs was also reduced by addition of cold pembrolizumab, whereas uptake in non-lymphoid tissues remained relatively constant throughout the entire study, indicating ^89^Zr-N-sucDf-pembrolizumab uptake in lymphoid tissues is PD-1-specific.

**Fig. 4.**
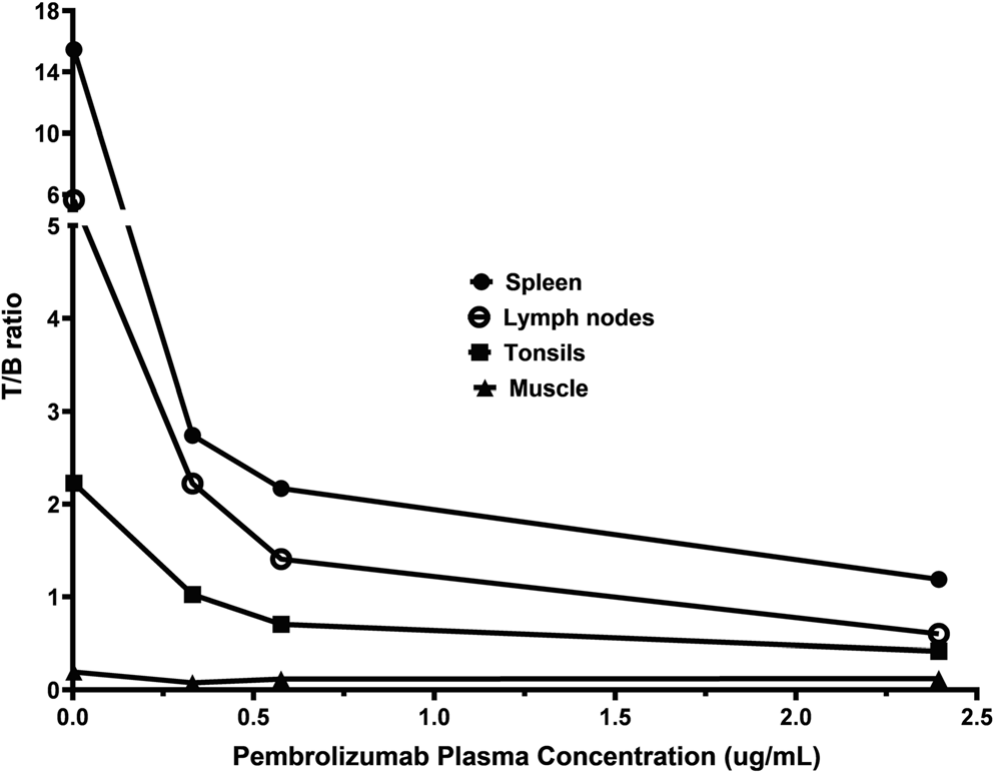
Dose-dependent decrease of ^89^Zr-N-sucDf-pembrolizumab tissue to blood pool (T/B) ratios in lymphoid tissues of cynomolgus monkeys at 5 days after administration of tracer and unlabeled pembrolizumab.

Plasma pembrolizumab concentrations are summarized in Table 2. The ratios of T/B in the spleen, lymph nodes, and tonsils at day 5 after tracer injection are associated with plasma concentration of pembrolizumab (Fig. 4). Following administration of ^89^Zr-N-sucDf-pembrolizumab to monkeys, dose-dependent decrease of T/B ratios in spleen, lymph nodes, and tonsils was observed, underscoring antibody penetration/distribution into these critical organs. The highest T/B ratios (15.45 for spleen, 5.64 for lymph nodes, 2.23 for tonsils) for ^89^Zr-N-sucDf-pembrolizumab in animal 1 was associated with the lowest pembrolizumab plasma concentration (≤ 0.003 μg/ml), while the lowest T/B ratios observed for animal 4 was associated with the highest pembrolizumab plasma concentration 2.395 μg/ml. The T/B ratios in non-target tissues like muscle remained relatively low with uptake independent to pembrolizumab concentration.

**Table 2.** Antibody plasma concentration (μg/ml) for ^89^Zr-N-sucDf-pembrolizumab in cynomolgus monkeys

Animal ID	Injected dose (mg/kg)	Day 0	Day 2	Day 5	Day 7
Animal 1	0.020	0.350	0.090	≤ 0.003^a^	≤ 0.003^a^
Animal 2	0.028	-	0.465	0.331	0.227
Animal 3	0.044	-	1.093	0.576	0.374
Animal 4	0.254	6.466	4.201	2.395	1.788

^a^Lower limit of quantitation (LLOQ) 0.003 μg/ml in plasma samples

The % ID for all organs considered over time, averaged across the 4 animals, are summarized in Supplementary Figure 2, and the estimations of human organ absorbed radiation from ^89^Zr-N-sucDf-pembrolizumab monkey data are given in Supplementary Table 1. For males, the highest absorbed dose was observed in the testes with 2.29 ± 1.03 mSv/MBq (*n* = 2). For females, the critical organ was found to be the spleen, 1.91 ± 0.24 mSv/MBq (*n* = 2). The liver (1.85 ± 0.62 mSv/MBq) and kidney (1.06 ± 0.26 mSv/MBq) absorbed doses are relatively higher compared with the absorbed doses from rodents [11], but in line with a typical ^89^Zr-labeled antibody [18, 19]. The whole-body effective dose for an adult human was estimated to be 0.88 ± 0.15 mSv/MBq, which is comparable to 0.515 ± 0.005 from mice and 0.540 ± 0.008 from rats, and is less than that received from typical abdominal CT scans [11]. This calculated dosimetry data is markedly similar to ^89^Zr-labeled cetuximab (0.60 ± 0.04 mSv/MBq) [18], which is within the average range of effective dose for ^89^Zr-labeled antibodies in humans [19].

## Discussion

The ^89^Zr-N-sucDf-pembrolizumab PET imaging results represent the first evaluation of the pharmacokinetics, distribution, and specific uptake of ^89^Zr-N-sucDf-pembrolizumab in cynomolgus monkeys. ^89^Zr-N-sucDf-pembrolizumab shows preferential uptake in the lymphoid tissues including the lymph nodes, spleen, and tonsils. In immune cell–rich lymphoid tissues (spleen, lymph nodes, and tonsils), tracer accumulation increased over time in all monkeys (Figs. 2 and 3). This is most likely due to radiolabeled pembrolizumab binding to PD-1-positive immune cells and other lymphocytes, which is consistent with published results [7, 20, 21]. Although the sample size in this study is small, we found the uptake of ^89^Zr-N-sucDf-pembrolizumab in lymphoid tissues decreases in proportion with the amount of co-injected PD-1 antibody mass (0.020–0.254 mg/kg) (Fig. 4). This data suggests that the tracer uptake in lymphoid tissues is mediated by binding to PD-1. This is consistent with clinical studies indicating that pembrolizumab peripheral target engagement in human is a function of administered dose and that pembrolizumab did not achieve full saturation until a dose of at least 1 mg/kg, which is higher than the doses administrated in the studies we report [21].

There was high retention of ^89^Zr-N-sucDf-pembrolizumab in the spleen, consistent with high spleen accumulation reported for anti-PD-1 antibody ^89^Zr-nivolumab in cynomolgus monkeys [20]. Compared with whole-body PET images of ^89^Zr-Nivolumab in patients with non-small-cell lung cancer [7], ^89^Zr-Nivolumab showed similar increasing spleen accumulation and decreasing concentration in the blood pool over time, a similar distribution in spleen and liver compared with the results with ^89^Zr-N-sucDf-pembrolizumab in this study.

The distribution of ^89^Zr-N-sucDf-pembrolizumab T/B ratios in non-target tissues including the liver, lung, kidney, muscle, and brain showed minimal change with co-administration of pembrolizumab, in agreement with non-displaceable tracer distribution, as expected for tissues with low expected levels of immune cells. Administration of unlabeled pembrolizumab resulted in a substantial increase of ^89^Zr-sucDf-pembrolizumab in the blood pool as a direct consequence of blocking PD-1-mediated radiotracer binding in T cell–rich lymphoid tissues, therefore increasing the amount of radiotracer available for circulation [22, 23]. In particular, the spleen is an organ that can act as an antigen sink due to the high abundance of PD-1-positive immune cells present and potentially influence the ability of a PD-1-binding ligand to reach the tumor microenvironment at lower doses of antibody. The ability of ^89^Zr-N-sucDf-pembrolizumab to image PD-1-expressing immune cells in patient tumors might rely on efficiently blocking this physiological uptake for consistent targeting.

The administered ^89^Zr-N-sucDf-pembrolizumab dose of approximately 10 MBq proved to be sufficient for adequate imaging up to 7 days after the injection while a conventional clinical PET/CT scanner with typical sensitivity was used. The PET images approached optimal signal-to-noise ratios in lymphoid tissues by day 5, with a further slight increase on day 7 (Fig. 3b, d). Compared with the ~ 37 MBq used in the cynomolgus monkey study with ^89^Zr-nivolumab [20], the ~ 10 MBq ^89^Zr dose used in the present study resulted in a lower radiation dose to the animals while preserving image quality.

The thymus is a central lymphoid organ that provides a microenvironment for T cell development, which is characterized by age-related atrophy. Compared with ^89^Zr-nivolumab PET images in cynomolgus monkeys, ^89^Zr-N-sucDf-pembrolizumab showed a very similar level and pattern of thymus uptake (Fig. 2), which decreased over time. The blockade was not observed with increasing pembrolizumab dose, suggesting that tracer distribution in the thymus was not PD-1-mediated. This was not unexpected, since there is a progressively decrease in the number of cortical lymphocytes during age-related thymus involution from about 4 or 5 years old in cynomolgus monkeys [24], and the monkeys in this study were over 7 years of age. Additionally, few studies have reported thymic uptake of radiopharmaceuticals assessing receptor expression on immune cells as a result of dramatic thymic function changes [25]; therefore, the ^89^Zr-N-sucDf-pembrolizumab retention in the thymus of monkeys over 5 years old is not considered to be a relevant organ to track the distribution of PD-1-positive immune cells.

Biodistribution at day 5 or 7 clearly indicated that ^89^Zr-N-sucDf-pembrolizumab targets PD-1-positive immune cells in lymphoid tissues, which exhibit a high T/B ratio when compared with clearance organs. Overall, the PET signal from the lymphoid tissues responding to ^89^Zr-N-sucDf-pembrolizumab is high enough to measure *in vivo* and well-delineated from other background tissues. In this study, we have correlated plasma concentrations of pembrolizumab as measured with an ECL-based ligand-binding assay to the PET signal-tracking PD-1-expressing immune cells, T/B ratios in lymphoid tissues appeared to decrease as a function of concentration and dose.

One limitation of our study is that we did not examine the PD-1 expression by immunohistochemical staining in lymphocyte tissues to correlate with tracer accumulation in tissues by PET imaging. However, the distribution in tissues known to be immune-cell rich and the reduction in tracer binding by excess pembrolizumab supports the PD-1-mediated uptake of ^89^Zr-N-sucDf-pembrolizumab in lymphocyte tissues. Due to the small sample size of monkeys, statistical analyses were not performed on these imaging data.

## Conclusions

^89^Zr-N-sucDf-pembrolizumab may be useful for tracking the distribution of PD-1-positive immune cells in cancer patients. Based on this data, human studies are warranted (a clinical molecular imaging study with ^89^Zr-N-sucDf-pembrolizumab is ongoing at the University Medical Center Groningen, The Netherlands. (ClinicalTrials.gov Identifier NCT02760225)), confirming the translatability of PD-1 imaging. Together, these studies support the rationale of using ^89^Zr-N-sucDf-pembrolizumab for the non-invasive assessment of tumor and whole-body PD-1 expression in cynomolgus monkeys and infection models.

## Electronic Supplementary Material

ESM 1 (DOCX 286 kb)
