# Supplementary material for: PET/CT Imaging of 89Zr-N-sucDf-Pembrolizumab in Healthy Cynomolgus Monkeys
Source: Mol Imaging Biol. 2020 Oct 26;23(2):250–9. doi: 10.1007/s11307-020-01558-w (PMC7910264; doi:10.1007/s11307-020-01558-w)
Supplement: Supplementary file 1 — (DOCX 286 kb) [file 11307_2020_1558_MOESM1_ESM.docx]

**Supplementary Material**

**Li et al.**

**Supplementary Figure 1**


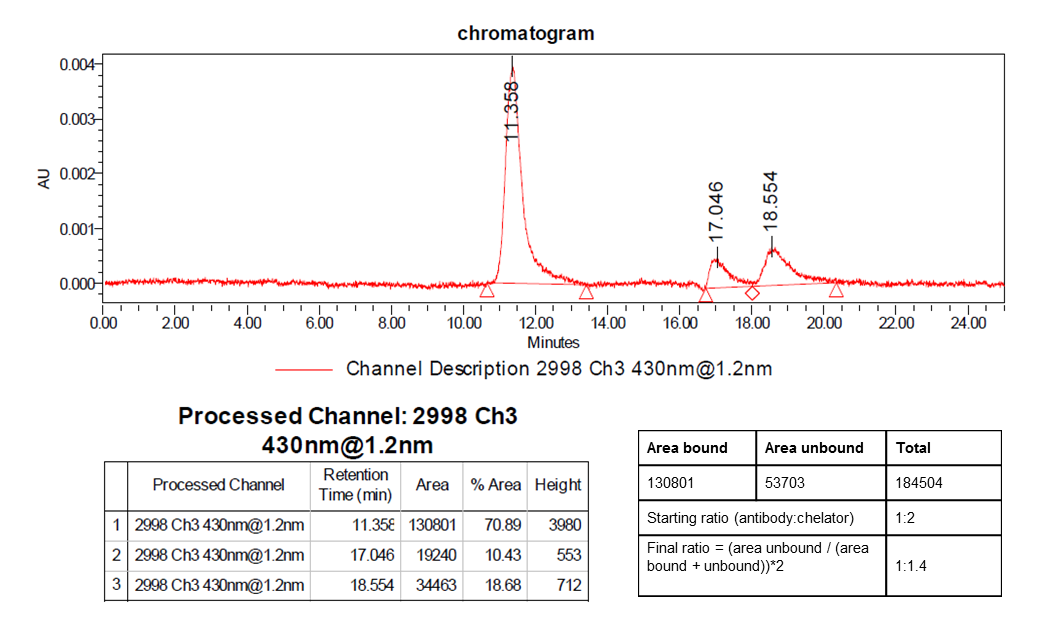


**Supplementary Figure 1.** HPLC chromatogram for determination of the final chelation ratio. Final chelation ratio is calculated by dividing the area for bound chelator by the total area (bound + unbound chelator), determined at 430 nm.

**Supplementary Figure 2**

***
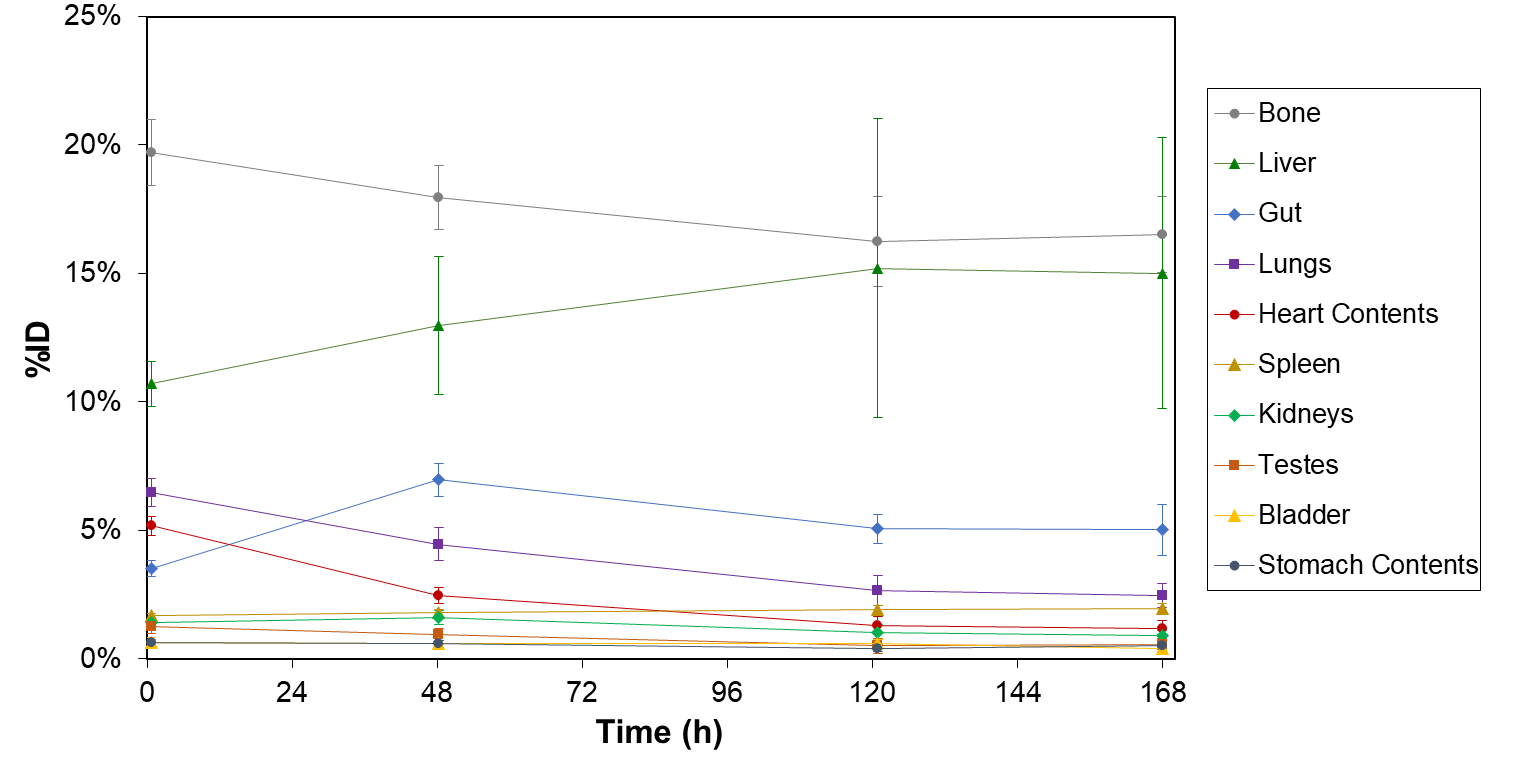
*Supplementary Figure 2.** Percent injected dose for all organs considered in dosimetry analysis. (error bars = standard error of the mean). ROIs were drawn independently for each scan in each animal.

**Supplementary Table 1**. **Absorbed Radiation Doses (mSv/MBq)**

| Organ | Animal 1 | Animal 2 | Animal 3 | Animal 4 | Average | St Dev |
| --- | --- | --- | --- | --- | --- | --- |
| Testes | 3.01 | 1.56 | N/A | N/A | 2.29 | 1.03 |
| Liver | 1.31 | 2.70 | 1.50 | 1.89 | 1.85 | 0.62 |
| Spleen | 1.76 | 1.75 | 1.74 | 2.08 | 1.83 | 0.17 |
| Red Marrow | 1.08 | 0.88 | 1.14 | 1.32 | 1.10 | 0.18 |
| Kidneys | 0.92 | 0.78 | 1.19 | 1.35 | 1.06 | 0.26 |
| Osteogenic Cells | 0.78 | 0.62 | 1.03 | 1.16 | 0.90 | 0.24 |
| Heart Wall | 0.95 | 0.70 | 0.94 | 0.97 | 0.89 | 0.12 |
| Lungs | 0.51 | 0.67 | 0.95 | 1.13 | 0.82 | 0.28 |
| Gallbladder Wall | 0.62 | 0.92 | 0.70 | 0.81 | 0.76 | 0.13 |
| Adrenals | 0.61 | 0.71 | 0.75 | 0.86 | 0.73 | 0.10 |
| LLI Wall | 0.59 | 0.44 | 0.91 | 0.81 | 0.69 | 0.21 |
| Pancreas | 0.58 | 0.65 | 0.70 | 0.79 | 0.68 | 0.09 |
| Stomach Wall | 0.44 | 0.51 | 0.70 | 0.73 | 0.60 | 0.14 |
| ULI Wall | 0.49 | 0.47 | 0.69 | 0.66 | 0.58 | 0.11 |
| Ovaries | N/A | N/A | 0.55 | 0.57 | 0.56 | 0.01 |
| Urinary Bladder Wall | 0.58 | 0.28 | 0.48 | 0.77 | 0.53 | 0.20 |
| Small Intestine | 0.44 | 0.39 | 0.55 | 0.56 | 0.48 | 0.08 |
| Uterus | N/A | N/A | 0.46 | 0.50 | 0.48 | 0.03 |
| Total Body | 0.37 | 0.36 | 0.46 | 0.50 | 0.42 | 0.07 |
| Thymus | 0.38 | 0.31 | 0.44 | 0.47 | 0.40 | 0.07 |
| Muscle | 0.31 | 0.27 | 0.37 | 0.40 | 0.34 | 0.06 |
| Breasts | 0.26 | 0.24 | 0.33 | 0.35 | 0.30 | 0.05 |
| Thyroid | 0.26 | 0.20 | 0.31 | 0.32 | 0.27 | 0.06 |
| Brain | 0.22 | 0.16 | 0.29 | 0.30 | 0.24 | 0.07 |
| Skin | 0.20 | 0.17 | 0.25 | 0.26 | 0.22 | 0.04 |
